# AMPK Mediates Muscle Mass Change But Not the Transition of Myosin Heavy Chain Isoforms during Unloading and Reloading of Skeletal Muscles in Mice

**DOI:** 10.3390/ijms19102954

**Published:** 2018-09-27

**Authors:** Tatsuro Egawa, Yoshitaka Ohno, Ayumi Goto, Shingo Yokoyama, Tatsuya Hayashi, Katsumasa Goto

**Affiliations:** 1 Department of Physiology, Graduate School of Health Sciences, Toyohashi SOZO University, Toyohashi, Aichi 440-8511, Japan; ayumi.goto8@gmail.com (A.G.); gotok@sepia.ocn.ne.jp (K.G.); 2 Laboratory of Sports and Exercise Medicine, Graduate School of Human and Environmental Studies, Kyoto University, Kyoto 606-8501, Japan; tatsuya@kuhp.kyoto-u.ac.jp; 3 Laboratory of Health and Exercise Sciences, Graduate School of Human and Environmental Studies, Kyoto University, Kyoto 606-8501, Japan; 4 Laboratory of Physiology, School of Health Sciences, Toyohashi SOZO University, Toyohashi, Aichi 440-8511, Japan; yohno@sozo.ac.jp (Y.O.); s-yokoyama@sozo.ac.jp (S.Y.)

**Keywords:** atrophy, regrowth, sirtuin 1 (SIRT1), peroxisome proliferator-activated receptor gamma coactivator 1-α (PGC1α), heat shock protein, fiber-type

## Abstract

5′AMP-activated protein kinase (AMPK) plays an important role in the regulation of skeletal muscle mass and fiber-type distribution. However, it is unclear whether AMPK is involved in muscle mass change or transition of myosin heavy chain (MyHC) isoforms in response to unloading or increased loading. Here, we checked whether AMPK controls muscle mass change and transition of MyHC isoforms during unloading and reloading using mice expressing a skeletal-muscle-specific dominant-negative AMPKα1 (AMPK-DN). Fourteen days of hindlimb unloading reduced the soleus muscle weight in wild-type and AMPK-DN mice, but reduction in the muscle mass was partly attenuated in AMPK-DN mice. There was no difference in the regrown muscle weight between the mice after 7 days of reloading, and there was concomitantly reduced AMPKα2 activity, however it was higher in AMPK-DN mice after 14 days reloading. No difference was observed between the mice in relation to the levels of slow-type MyHC I, fast-type MyHC IIa/x, and MyHC IIb isoforms following unloading and reloading. The levels of 72-kDa heat-shock protein, which preserves muscle mass, increased in AMPK-DN-mice. Our results indicate that AMPK mediates the progress of atrophy during unloading and regrowth of atrophied muscles following reloading, but it does not influence the transition of MyHC isoforms.

## 1. Introduction

The skeletal muscle is the largest organ in the body and plays a crucial role in metabolism. Loss of skeletal muscle function and mass leads to disorders such as sarcopenia and insulin resistance [1]. Muscle loading is a vital process in the regulation of skeletal muscle properties. Increased loading induced by mechanical stretch or strength exercises leads to muscle hypertrophy and regrowth of atrophied skeletal muscles [2,3,4]. By contrast, unloading, as well as inactivity, causes skeletal muscle atrophy, especially that of the antigravitational slow-twitch muscles [5,6].

5′AMP-activated protein kinase (AMPK) is a central regulator of cellular metabolism and energy homeostasis in mammalian tissues. AMPK plays an important role in the regulation of skeletal muscle mass; AMPK inhibits hypertrophy of skeletal muscle cells [7,8,9] and rodent skeletal muscle [7,10], and AMPK activity negatively correlates the degree of hypertrophy in rat skeletal muscle [11,12]. We have previously shown that AMPK regulates unloading-induced skeletal muscle atrophy [13]. However, it remains unclear whether AMPK plays a role in the regrowth of atrophied skeletal muscles in response to increased loading.

The skeletal muscle fibers in mammals can be roughly divided into slow- and fast-twitch types, which are further classified into type I, type IIa, type IIx, and type IIb. The four muscle fibers, respectively, contain protein isoforms of myosin heavy chain (MyHC) I, IIa, IIx, and IIb [14]. The slow-to-fast transition of MyHC isoforms is observed in unloading-associated atrophied slow soleus muscles [4,15]. In this regard, AMPK is a potential regulator of skeletal muscle fiber-type distribution. Training-induced increases in MyHC IIa/x isoforms is attenuated in AMPKα2-deficient mice [16]. Chronic administration of an AMPK activator promotes a switch to type I fibers in the skeletal muscles of rodents [16,17,18]. Furthermore, AMPK triggers oxidative adaptation and is involved in training-induced fiber-type shift [17]. However, to date, it remains unclear whether AMPK is involved in the transition of MyHC isoforms during unloading and reloading.

AMPK controls skeletal muscle plasticity through a variety of molecular responses. Interaction of AMPK with sirtuin 1 (SIRT1) and peroxisome proliferator-activated receptor gamma coactivator 1-α (PGC1α) comprises a pivotal regulatory network in metabolic homeostasis [19]. In terms of muscle mass regulation, the AMPK–SIRT1 axis acts as a sensor of nutrient availability and regulates muscle development [20], and pharmacological AMPK activation inhibits muscle cell growth through a PGC1α-dependent mechanism [21]. In addition, we recently showed that the interaction of AMPK with 72-kDa heat-shock protein (HSP72) is associated with hypertrophy as well as atrophy of skeletal muscles [9,13]. Although AMPK’s regulation of muscle mass is thus clear, no previous study, to our knowledge, has examined the association between AMPK and SIRT1, PGC1α, and HSP72 during unloading-induced atrophy or regrowth following atrophy.

**Figure 1:**
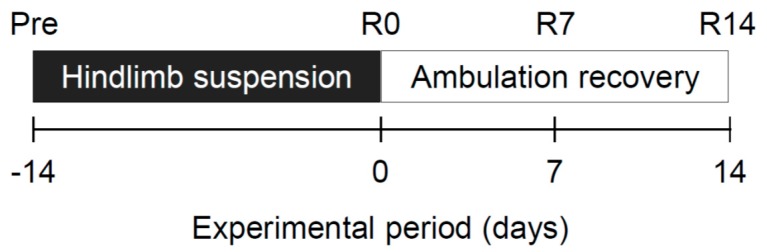
Summary of the experimental protocol. The hindlimbs of both dominant-negative mutant of AMPK (AMPK-DN) and wild-type littermate (WT) mice were continuously suspended for 14 days. After 14 days, the mice were allowed ambulation recovery. Pre: before hindlimb suspension; R0, R7, and R14: 0, 7, and 14 days after ambulation recovery; *n* = 8 per group.

Here, we investigated the role of AMPK in the changes in muscle mass and transition of MyHC isoforms, and its associated molecular responses during unloading and reloading. We performed 14-day hindlimb suspension and 14-day ambulation recovery procedures with wild-type littermate (WT) mice and transgenic mice that overexpressed a muscle-specific dominant-negative mutant of AMPKα1 (AMPK-DN) (Figure 1) [22]. AMPK is a heterotrimeric kinase, consisting of a catalytic α-subunit and two regulatory subunits, β and γ. Two distinct α-isoforms (α1 and α2) exist in skeletal muscle. These mice exhibit almost complete depletion in AMPKα2 activity and moderate depletion in AMPKα1 activity [22,23,24,25].

## 2. Results

### 2.1. 5′AMP-Activated Protein Kinase (AMPK) Activity

**Figure 2:**
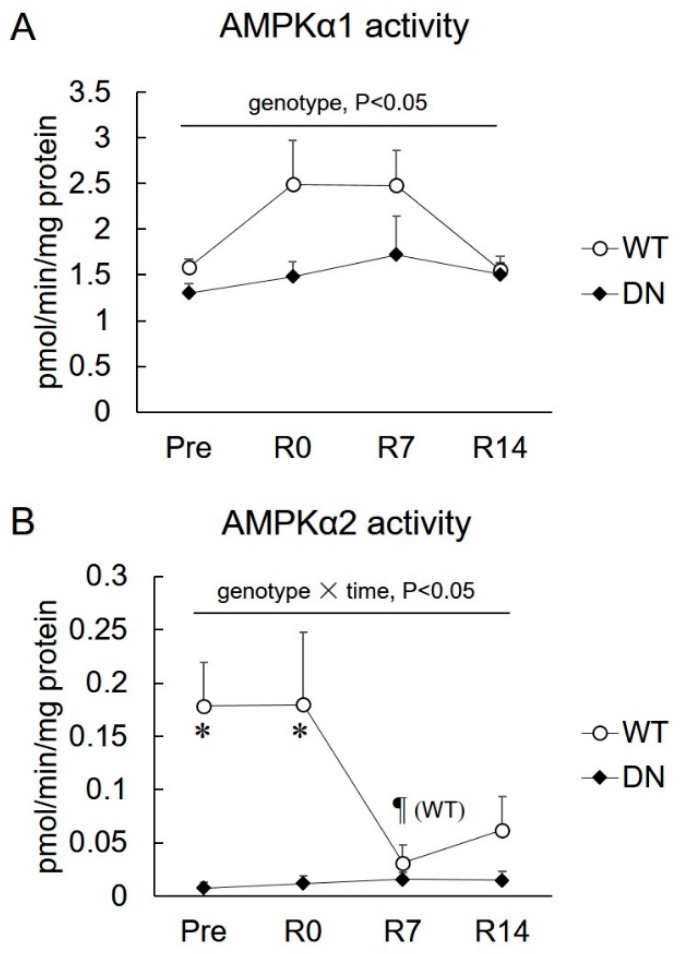
Changes in the isoform-specific 5′AMP-activated protein kinase (AMPK) activity after 14 days of hindlimb unloading, and at 0, 7, and 14 days of reloading. (**A**) AMPKα1 activity. (**B**) AMPKα2 activity. Values are means ± SE; *n* = 7–8 per group. Statistical results of two-way ANOVA (genotype, time, and genotype × time) are described in the Figure. *, significant difference between genotypes at same time point. ¶, significant difference from R0 independent of genotype, unless special mention in Figure.

Changes in isoform-specific AMPK activities during unloading and reloading are shown in Figure 2. AMPKα1 activity was lower in AMPK-DN mice than that of WT mice during the overall experimental period (genotype effect, *p* < 0.05), but there was no time effect in AMPKα1 activity (Figure 2A). AMPKα2 activity was almost completely suppressed in AMPK-DN mice compared with that in WT mice during the overall experimental period (genotype effect, *p* < 0.05) (Figure 2B). AMPKα2 activity decreased in response to 7 days of reloading, and remained suppressed after 14 days of reloading (Figure 2B).

### 2.2. Body Weight and Soleus Muscle Weight

**Figure 3:**
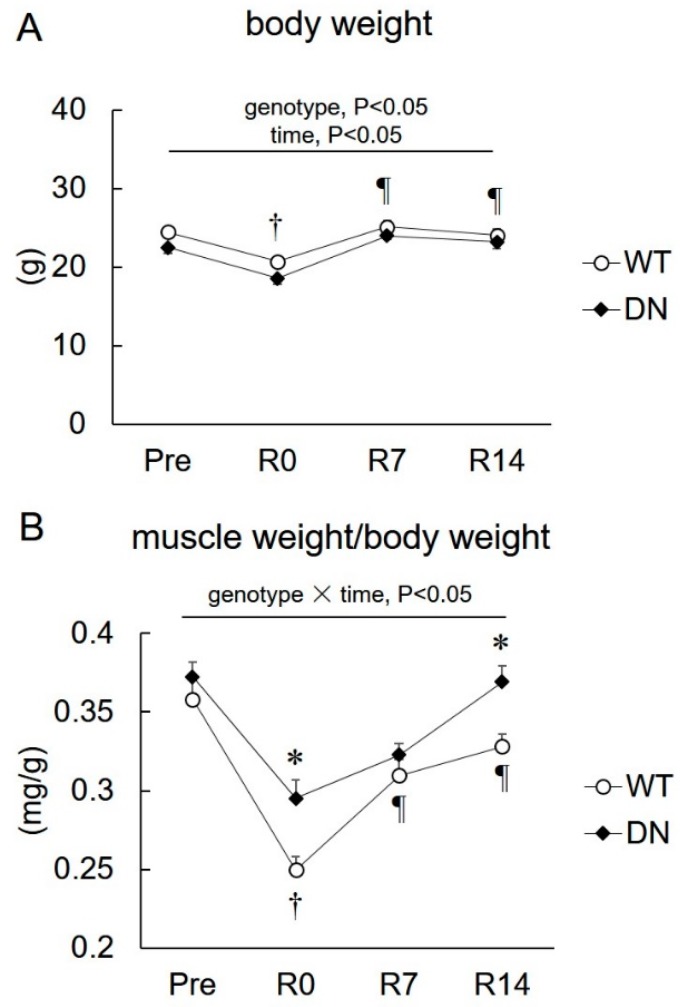
Changes in the body weight and soleus weight after 14 days of hindlimb unloading, and at 0, 7, and 14 days of reloading. (**A**) Body weight. (**B**) Relative soleus weight to body weight. Values are means ± SE; *n* = 8 per group. Statistical results of two-way ANOVA (genotype, time, and genotype × time) are described in the Figure. *, significant difference between genotypes at same time point. †, significant difference from Pre independent of genotype. ¶, significant difference from R0 independent of genotype.

Changes in body weight and soleus muscle weight relative to the body weight during unloading and reloading are shown in Figure 3. The body weight was lower in AMPK-DN mice than that in WT mice during the overall experimental period (genotype effect, *p* < 0.05) (Figure 3A). The body weight of both mice decreased in response to 14 days of unloading and was restored after 7 days of reloading (Figure 3A). The soleus muscle weight relative to the body weight also decreased following the 14-day unloading, and recovered after 7 and 14 days of reloading for both groups, but the reduction was attenuated in AMPK-DN mice compared with that in WT mice (Figure 3B). There was no difference in the muscle weight of both mice groups after 7 days of reloading, but it was higher in AMPK-DN mice after 14 days reloading (Figure 3B).

### 2.3. Levels of Slow- and Fast-Type Myosin Heavy Chain (MyHC) Isoforms

**Figure 4:**
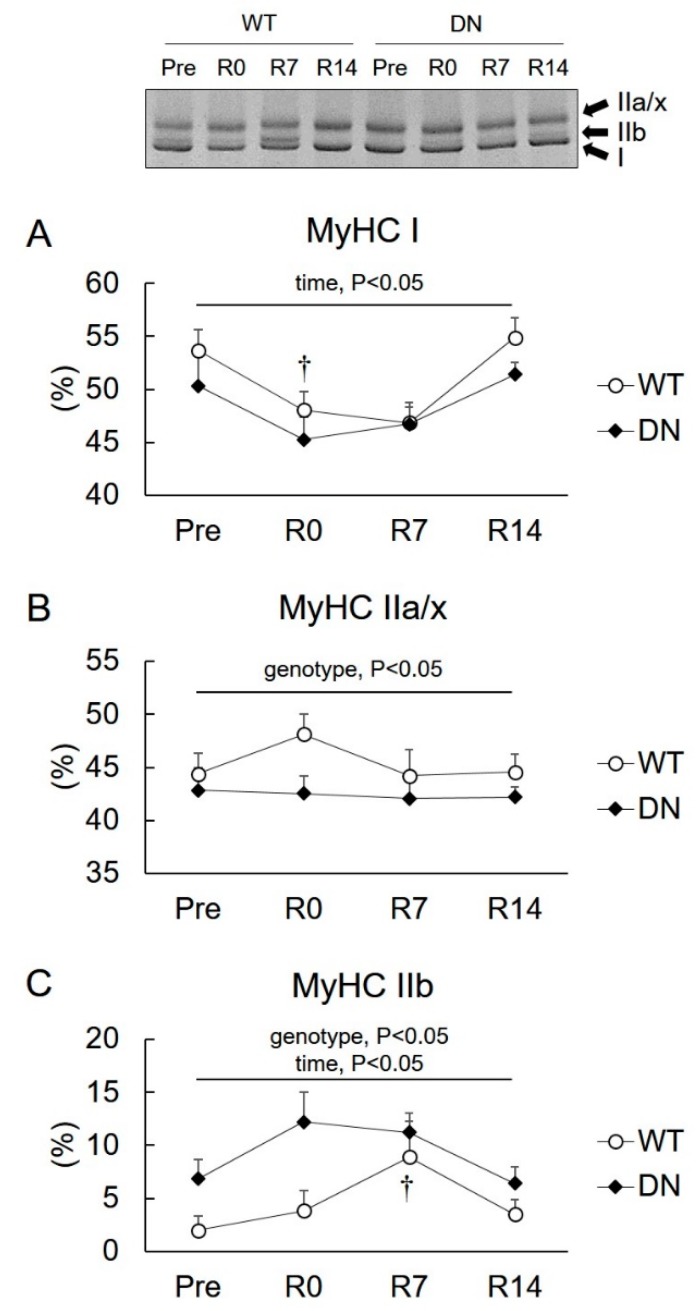
Changes in relative levels of myosin heavy chain (MyHC) isoforms in the soleus muscles after 14 days of hindlimb unloading, and at 0, 7, and 14 days of reloading. (**A**) MyHC I. (**B**) MyHC IIa/x. (**C**) MyHC IIb. Representative image is shown. Values are means ± SE; *n* = 8 per group. Statistical results of two-way ANOVA (genotype, time, and genotype × time) are described in the Figure. †, significant difference from Pre independent of genotype.

We examined the levels of slow-type I and fast-type IIa, IIx, and IIb MyHC isoforms in the soleus muscle (Figure 4). Owing to a technical limitation, IIa and IIx MyHC phenotypes were expressed as IIa/x. MyHC I levels decreased following 14 days of unloading, but there was no difference between their levels in WT and AMPK-DN mice (Figure 4A). The MyHC IIa/x level was lower in AMPK-DN mice than that in WT mice during the overall experimental period (genotype effect, *p* < 0.05), but no change was observed following unloading and reloading (Figure 3B). The MyHC IIb level was higher in AMPK-DN mice than that in WT mice during the overall experimental period (genotype effect, *p* < 0.05) (Figure 4C). The levels did not change by unloading, but increased after seven days of reloading in both mice groups (Figure 4C).

### 2.4. Sirtuin 1 (SIRT1) Activity and Peroxisome Proliferator-Activated Receptor Gamma Coactivator 1-α (PGC1α) mRNA Levels

**Figure 5:**
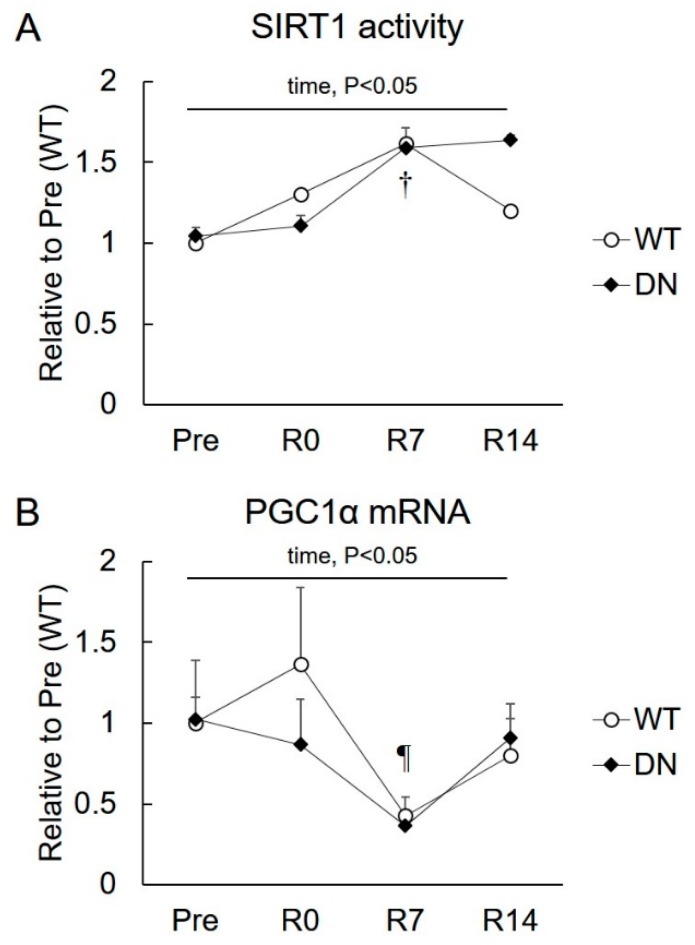
Changes in sirtuin 1 (SIRT1) activity and peroxisome proliferator-activated receptor gamma coactivator 1-alpha (PGC1α) mRNA expression after 14 days of hindlimb unloading, and at 0, 7, and 14 days of reloading. (**A**) SIRT1 activity; *n* = 3 per group. (**B**) PGC1α mRNA; *n* = 8 per group. Values are means ± SE. Statistical results of two-way ANOVA (genotype, time, and genotype × time) are described in the Figure. †, significant difference from Pre independent of genotype. ¶, significant difference from R0 independent of genotype.

To clarify the role of SIRT1 and PGC1α in the changes in the AMPK-mediated phenotype during muscle mass change, SIRT1 activity and PGC1α mRNA levels were examined (Figure 5). SIRT1 activity increased following 7 days of reloading, but it was similar in the WT and AMPK-DN mice (Figure 5A). No difference was observed in the PGC1α mRNA level between WT and AMPK-DN mice during the overall experimental period (Figure 5B). The PGC1α mRNA level was not changed after 14 days of unloading, but it decreased in response to 7 days of reloading in both mice groups (Figure 5B).

### 2.5. 72-kDa Heat-Shock Protein (HSP72) Levels

**Figure 6:**
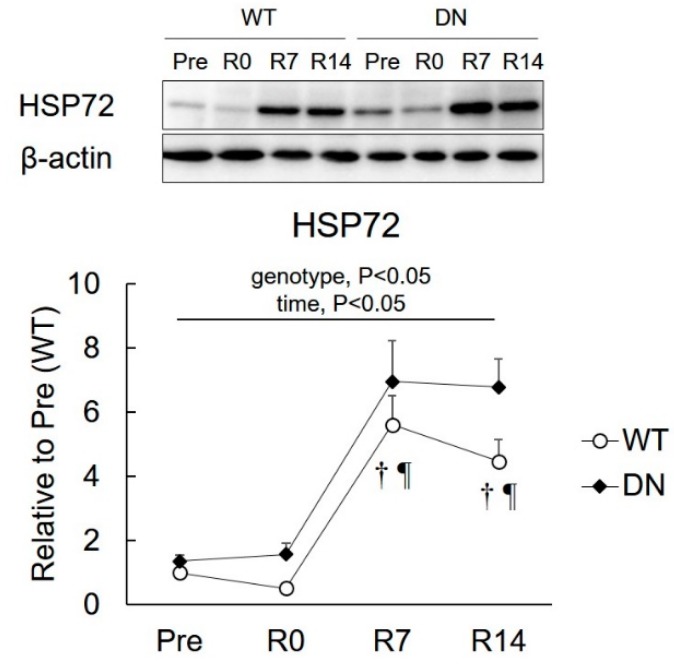
Changes in the 72-kDa heat shock protein (HSP72) expression after 14 days of hindlimb unloading, and at 0, 7, and 14 days of reloading. Representative immunoblots are shown. Values are means ± SE. Statistical results of two-way ANOVA (genotype, time, and genotype × time) are described in the Figure. †, significant difference from Pre independent of genotype. ¶, significant difference from R0 independent of genotype.

HSP72 levels were determined to examine the relationship between HSP72 and AMPK-mediated skeletal muscle atrophy and regrowth (Figure 6). The HSP72 level was higher in AMPK-DN mice than that in WT mice during the overall experimental period (genotype effect, *p* < 0.05) (Figure 6B). HSP72 levels increased following 7 days of reloading, which remained unchanged throughout the 14 days of reloading in both mice groups (Figure 6).

## 3. Discussion

The present study reports the following novel findings with respect to the role of AMPK in muscle mass change and fiber-type shift during unloading and reloading. First, AMPKα2 activity is suppressed in response to the reloading procedure (Figure 2). Second, the regrowth of soleus muscle weight in response to reloading was accelerated in AMPK-DN mice after seven days reloading (Figure 3). Third, AMPK-DN mice showed a higher proportion of MyHC IIb than WT mice, and the slow-to-fast transition of MyHC isoforms was identical in WT and AMPK-DN mice (Figure 4). Fourth, no difference was observed between the mice in response to unloading- and reloading-induced changes of SIRT1 activity and PGC1α mRNA levels (Figure 5). Fifth, AMPK-DN mice showed higher levels of HSP72 (Figure 6) in the soleus muscles than WT mice.

It is accepted that diminished loading leads to skeletal muscle atrophy, and increased loading following unloading induces regrowth [2,3,4,5,6]. Fourteen days of hindlimb unloading showed a 30% decrease in the soleus muscle mass of WT mice, whereas it was attenuated in AMPK-DN mice (Figure 3A). Such atrophic responses of the soleus muscles were in accordance with those seen in our previous study, which used the same procedures [13]. Our findings did not show the upregulation of AMPKα1 and α2 activity in response to 14 days of unloading (Figure 2). A previous study reported that AMPK signaling was activated at the early phase (three days) of unloading and returned to the basal level at 7 days [26]. This may be why we could not detect the upregulation of AMPK activity after 14 days of unloading.

The difference in muscle weight disappeared following seven days reloading. Interestingly, the difference was expanded again after 14 days reloading (Figure 3B). This suggests that the lack of AMPK activity promotes regrowth of atrophied skeletal muscles, especially in the latter phase of recovery. AMPK is known to be a negative regulator of muscle hypertrophy, and this is supported by our findings that AMPKα2 activity is greatly suppressed during the reloading (hypertrophy) phase (Figure 2B). A previous report has shown that AMPK phosphorylation was suppressed by seven days of reloading following unloading in mouse heart muscle [27]. Therefore, reduction of AMPK activity (mainly α2) might contribute to progress in skeletal muscle regrowth. To our knowledge, this is the first study to report on the association of AMPK with regrowth from unloading-induced atrophy of skeletal muscles.

The soleus muscles of adult mice have high levels of slow-type I and fast-type IIa MyHC, and low levels of fast-type IIx or IIb MyHC [4]. Unloading results in a slow-to-fast transition of MyHC isoforms [4,15], whereas reloading reverses it [28]. We saw that 14 days of unloading decreased MyHC I levels and tended to increase MyHC IIa and IIb levels (Figure 4), suggesting that a slow-to-fast transition of MyHC isoforms occurred. In addition, the 14 days of reloading induced a fast-to-slow transition of MyHC. However, the transition occurred identically in WT and AMPK-DN mice. This suggests that AMPK does not play a role in unloading- and reloading-induced transition of MyHC isoforms. On the other hand, we found that AMPK-DN mice exhibited an increased proportion of MyHC IIb (Figure 4C) and decreased proportion of MyHC IIa/x (Figure 4B). These results indicate that lack of AMPK activity (mainly α2) leads to slow-to-fast transition of muscle fiber type, suggesting that AMPK is associated with a regulation of skeletal muscle fiber-type distribution, as has been suggested [16,17,18].

SIRT1 is a key modulator of cell proliferation, hormone response, stress response, apoptosis, and cell metabolism [29]. AMPK and SIRT1 both regulate each other and share many common target molecules, including PGC1α [30]. Previous studies have shown that AMPK regulates muscle formation through SIRT1- or PGC1α-dependent mechanisms [20,21]. Moreover, SIRT1 and PGC1α both protect skeletal muscles from denervation-induced atrophy [31,32], suggesting that they both are important regulators of skeletal muscle mass. Here, no difference was observed between the WT and AMPK-DN mice in response to the unloading- and reloading-induced change of PGC1α mRNA levels (Figure 5B), and no significant difference in SIRT1 activity between the mice (Figure 5A). However, AMPK-DN mice exhibited higher SIRT1 activity after 14 days of reloading compared with that in WT mice, although this was not statistically significant. Moreover, considering that PGC1α expression and activity are controlled by posttranslational modifications [33], more detailed experiments are needed to make clear the involvement of SIRT1 and PGC1α in AMPK-mediated regrowth of atrophied muscle.

HSP72 is one of the most prominent members of the HSP family. Previous studies have shown that HSP72 levels increase under hypertrophic conditions [34,35], whereas they decrease under atrophic conditions [13,29]. Moreover, we have previously shown that an HSP-dependent mechanism underlies the AMPK-mediated inhibition of skeletal muscle hypertrophy [9]. Thus, HSP72 possibly plays an important role in muscle mass regulation. In the present study, we found that the HSP72 levels in the soleus muscles of AMPK-DN mice were higher than those in WT mice (Figure 6). In this regard, it has been shown that overexpressing HSP72 in transgenic mice prevents immobilization-induced skeletal muscle atrophy [36], and improves skeletal muscle recovery from unloading-induced atrophy [37]. We therefore hypothesized that the protection from atrophy and acceleration of regrowth of skeletal muscles in AMPK-DN mice was partly attributable to the high levels of HSP72.

Previously, we have shown that AMPK controlled hypertrophy and atrophy of skeletal muscle through protein degradation systems [9]. In addition, it has been shown that AMPK maintained muscle cell size through protein synthesis pathways [10]. Skeletal muscle mass is ultimately determined by balancing protein synthesis and degradation, and thus our findings that the suppression of AMPK activity attenuated unloading-induced atrophy and accelerated regrowth of skeletal muscle are probably attributed to changes in protein turnover systems, for example, mammalian target of rapamycin signaling, autophagy, and the ubiquitin-proteasome system. Therefore, further investigations measuring protein turnover systems are required to validate AMPK-mediated muscle mass regulation during unloading and reloading.

In conclusion, our current results indicate that AMPK mediates the progress of atrophy of skeletal muscles during unloading, and regrowth of atrophied muscles following reloading. To the best of our knowledge, this is the first report to show the effect of AMPK on skeletal muscle adaptations following recovery from unloading-induced atrophy. Our findings contribute to understanding the complex molecular responses during loading-associated skeletal muscle adaptations.

## 4. Materials and Methods

### 4.1. Animals

Transgenic mice expressing a dominant-negative mutant of AMPKα1 in the skeletal muscles [22] were purchased from the Laboratory Animal Resource Bank at the National Institute of Biomedical Innovation (Resource No. nbio085, Osaka, Japan). Male 12–16 week old AMPK-DN mice (*n* = 32) and WT littermate mice (*n* = 32) with C57BL/6NCr background were used. All mice were housed in an animal room maintained at 22–24 °C with a 12-h light–dark cycle, and were fed a standard laboratory diet with water given ad libitum. All animal-related protocols were performed in accordance with the Guide for the Care and Use of Laboratory Animals as adopted and promulgated by the National Institutes of Health (Bethesda, MD, USA), and were approved by the Animal Use Committee at Toyohashi SOZO University (A2012002, approved 7 August 2012; A2013003, approved 6 August 2013; and A2014003, approved 27 August 2014).

### 4.2. Procedure of Hindlimb Suspension and Ambulation Recovery

The hindlimbs of both AMPK-DN and WT mice were continuously suspended for 14 days as described previously [38]. After 14 days, the mice were allowed ambulation recovery. Eight mice of each strain were killed at baseline (untreated pre-experimental control: Pre), and at 0 (R0), 7 (R7) and 14 (R14) days of ambulation recovery (Figure 1). Their soleus muscles were dissected under anesthesia with intraperitoneal injection of sodium pentobarbital (50 mg/kg). The muscles were trimmed of excess fat and connective tissues, weighed, frozen in liquid nitrogen, and stored at −80 °C. The left soleus muscle was cross-sectionally sliced into halves at the mid-belly region, and the proximal half of the left soleus muscle was immediately frozen in 2-methylbutane cooled with liquid nitrogen, and stored at −80 °C for immunohistochemical analyses. The distal half of the left soleus muscle was used for real-time RT-PCR analysis, and the right soleus muscle was used for western blotting.

### 4.3. Sample Preparation and Western Blotting

Sample preparation and western blotting were performed as described previously [34,39]. Briefly, the muscles were homogenized in ice-cold lysis buffer (CelLytic MT, Sigma–Aldrich, St. Louis, MO, USA) containing a protease/phosphatase inhibitor (5872, Cell Signaling Technology, Danvers, MA, USA). The homogenates were then centrifuged at 16,000× *g* for 15 min at 4 °C. The supernatant was collected and solubilized in Laemmli’s sample buffer containing mercaptoethanol and was then boiled. Protein samples (10 μg) were separated by SDS-PAGE using a 10% polyacrylamide gel, after which the proteins were transferred onto polyvinylidene difluoride membranes. Next, the membranes were blocked for 1 h at room temperature in Blocking One-P (Nacalai Tesque, Kyoto, Japan), and then incubated overnight at 4 °C with primary antibodies: HSP72 (ADI-SPA-812, Enzo Life Sciences, Farmingdale, NY, USA), and β-actin (4967, Cell Signaling Technology); and diluted in Tris-buffered saline with 0.1% Tween 20 (TBS-T). The membranes were then washed with TBS-T and treated with anti-rabbit IgG (7074, Cell Signaling Technology) for 1 h at room temperature. After the final wash with TBS-T, protein bands were visualized using chemiluminescence (Wako Pure Chemical Industries, Osaka, Japan). The intensity of the signals was quantified using ImageJ (National Institutes of Health, Bethesda, MD, USA). The level of β-actin was evaluated as an internal control.

### 4.4. Real-Time RT-PCR Analyses

Real-time RT-PCR analyses were performed as was described previously [35]. Briefly, total RNA was extracted from muscles using the miRNeasy Mini kit (Qiagen, Hilden, Germany) according to the manufacturer’s protocol. For the detection of mRNA, the RNA was reverse-transcribed to cDNA using PrimeScript RT Master Mix (Takara Bio, Otsu, Japan), and then synthesized cDNA was applied to real-time RT-PCR (Thermal Cycler Dice Real Time System IIMRQ, Takara Bio) using Takara SYBR Premix Ex Taq II (Takara Bio). Relative fold change of expression was calculated by the comparative CT method. To normalize the amount of total RNA present in each reaction, S18 ribosomal RNA (18S rRNA) was used as an internal standard. The following primers were used: PGC1α (Ppargc1a), 5′-GCTGCATGGTTCTGAGTGCTAAG-3′ (forward) and 5′-AGCCGTGACCACTGACAACGAG-3′ (reverse); 18S rRNA, 3′-ACTCAACACGGGAAACCTCA-5′ (forward) and 3′-AACCAGACAAATCGCTCCAC-5′ (reverse).

### 4.5. Myosin Heavy Chain (MyHC) Isoform Detection

Analysis of MyHC isoform (I, IIa, IIx, and IIb) levels was performed using a previously described method, albeit with a modification [40,41]. Briefly, the homogenate sample proteins (5 μg) were separated by SDS-PAGE using a 7% polyacrylamide gel at 120 V for 19 h in a temperature-controlled chamber at 4 °C. After electrophoresis, the gels were stained with Oriole™ Fluorescent Gel Stain (Bio-Rad Laboratories, Hercules, CA, USA). The gels were visualized using Light-Capture (AE-6971, ATTO Corporation, Tokyo, Japan) and analyzed using ImageJ.

### 4.6. Sirtuin 1 (SIRT1) Activity Assay

SIRT1 activity was determined using the SIRT1 Fluorometric Assay Kit (AS-72155, AnaSpec, Fremont, CA, USA) according to the manufacturer’s instructions. Fluorescence was measured using a fluorometric reader (Fluoroskan FL, ThermoFisher Scientific, Waltham, MA, USA) with excitation at 490 nm and emission at 520 nm.

### 4.7. 5′AMP-Activated Protein Kinase (AMPK) Activity Assay

The kinase activities of α1-containing AMPK complex (AMPKα1) and α2-containing AMPK complex (AMPKα2) were measured as described previously [39]. The supernatants from the muscle homogenates (50 μg protein) were incubated with either the anti-α1 or -α2 antibody [42] and Protein A Sepharose beads (Amersham Biosciences, Uppsala, Sweden) at 4 °C overnight. The beads were subjected to the kinase reaction using the SAMS peptide as a substrate.

### 4.8. Statistical Analyses

All values were expressed as means ± SE. Statistical significance was analyzed using two-way analysis of variance (ANOVA), with genotype (WT and AMPK-DN) and time (Pre, R0, R7, and R14) as the main factors. If there was significant time effect, post hoc multiple-comparison tests were performed among groups (Pre, R0, R7, and R14). If there were any significant interactions (genotype × times), post hoc simple-effects tests were performed. Post hoc analyses were conducted with Tukey–Kramer’s test. The differences between groups were considered statistically significant at *p* < 0.05.

## Abbreviations

AMPK	5′AMP-activated protein kinase
AMPK-DN	dominant-negative mutant of AMPKα1
AMPKα1	α1-containing AMPK complex
AMPKα2	α2-containing AMPK complex
ANOVA	analysis of variance
HSP72	72-kDa heat shock protein
MyHC	myosin heavy chain
PGC1α	peroxisome proliferator-activated receptor gamma coactivator 1-alpha
TBS-T	Tris buffered saline with 0.1% Tween 20
